# Left ventricular ejection fraction and right atrial diameter are associated with deep regional CBF in arteriosclerotic cerebral small vessel disease

**DOI:** 10.1186/s12883-021-02096-w

**Published:** 2021-02-11

**Authors:** Xiaodong Chen, Danli Lu, Ning Guo, Zhuang Kang, Ke Zhang, Jihui Wang, Xuejiao Men, Zhengqi Lu, Wei Qiu

**Affiliations:** 1 Department of Neurology, The Third Affiliated Hospital of Sun Yat-sen University, Guangzhou, 510630 China; 2 Department of Radiology, The Third Affiliated Hospital of Sun Yat-sen University, Guangzhou, 510630 China; 3 Department of Psychiatry, The Third Affiliated Hospital of Sun Yat-sen University, Guangzhou, 510630 China

**Keywords:** Cerebral small vessel disease, Arteriolosclerosis, Cerebral blood flow, CSVD burden, Echocardiography

## Abstract

**Background:**

Systemic cardiac hypoperfusion is a well-acknowledged contributor to ischemic leukoencephalopathy. However, it has remained elusive how atherosclerosis-mediated cardiac remodelling modifies cerebral perfusion homeostasis as well as neuroimaging burden in cerebral small vessel disease (CSVD) development.

**Methods:**

This retrospective study identified 103 arteriosclerotic CSVD (aCSVD) patients (CSVD burden^low^ 0 ~ 1, *n* = 61 and CSVD burden^high^ 2 ~ 4, *n* = 42) from Sep. 2017 to Dec. 2019 who underwent transthoracic echocardiography(*n* = 81), structural magnetic resonance imaging and arterial spin labelling (ASL). Total CSVD burden was graded according to the ordinal “small vessel disease” rating score (0–4). We investigated the univariate and multivariate linear regression of mean deep regional cerebral blood flow (CBF) as well as logistic regression analysis of CSVD burden^high^.

**Results:**

Right atrial diameter (*B coefficient*, − 0.289; *95% CI*, − 0.578 to − 0.001; *P* = 0.049) and left ventricular ejection fraction (*B coefficient*, 32.555; *95% CI*, 7.399 to 57.711; *P* = 0.012) were independently associated with deep regional CBF in aCSVD patients. Binary logistic regression analysis demonstrated decreased deep regional CBF (*OR* 0.894; *95% CI* 0.811–0.985; *P* = 0.024) was independently associated with higher CSVD burden after adjusted for clinical confounders. Multivariate receiver operating characteristics curve integrating clinical risk factors, mean deep CBF and echocardiographic parameters showed predictive significance for CSVD burden^high^ diagnosis (area under curve = 84.25, *95% CI* 74.86–93.65%, *P* < 0.0001).

**Conclusion:**

The interrelationship of “cardiac -deep regional CBF-neuroimaging burden” reinforces the importance and prognostic significance of echocardiographic and cerebral hemodynamic assessment in CSVD early-warning.

**Supplementary Information:**

The online version contains supplementary material available at 10.1186/s12883-021-02096-w.

## Background

Cerebral small vessel disease (CSVD) is a spectrum of cerebrovascular diseases attributed to arteriolosclerosis, genetic inheritance, infection, autoimmune inflammation, venous collagenosis and other secondary aetiologies such as radiation [1]. Arteriosclerotic CSVD (aCSVD) is the most prevalent category in the elderly and contributes to high worldwide disease burden from stroke [2] and vascular dementia [3]. Advanced age and hypertension are the most evidenced epidemiological risk factors [4] and individualized therapy based on modifiable cardiovascular risk factors is the most widely accepted therapeutic and preventive strategy in clinical practice. The pathogenesis of aCSVD remains poorly illustrated and chronic cerebral hypoperfusion secondary to arteriolosclerosis is one of the attributable mechanism [1]. Most studies concerned with the relationship between cerebral perfusion and CSVD are based on white matter hyperintensity (WMH) development [5, 6]. Though persistent cerebral perfusion has been considered as an attributable mechanism of WMH, a longitudinal quantitative neuroimaging study reveals that relative cerebral blood flow (CBF) change has no significant correlation with WMH progression [7]. Notably, aforementioned studies mainly focus on global CBF alteration but not deep regional CBF which represents the circulation perfusion of parenchymal small vessels.

It is acknowledged that chronic systemic hypoperfusion can contribute to WMH and recent clinical investigations have revealed heart involved mechanisms including cerebral hypoperfusion [8] or sympathetic overactivity in CSVD development [9–11]. Thereinto, left ventricular ejection fraction (LVEF), a well-established cardiac perfusion indicator, reduces considerably in CSVD patients with dementia [12]. However, no considerable effect of LVEF on white matter microstructure damage and deterioration of CBF are observed in healthy middle-aged cohort [13]. Actually, WMH is predisposed to lower CBF perfusion in patients with heart failure but not in patients with normal cardiac function [14]. Collectively, how atherosclerosis mediated cardiac remodelling modifies deep regional CBF and neuroimaging burden in aCSVD patients with normal cardiac function remains unknown. We aimed to preliminarily investigate the interrelationship among echocardiographic parameters, deep regional CBF and CSVD neuroimaging markers in aCSVD patients absent from heart failure.

## Methods

### Study population

**Fig. 1:**
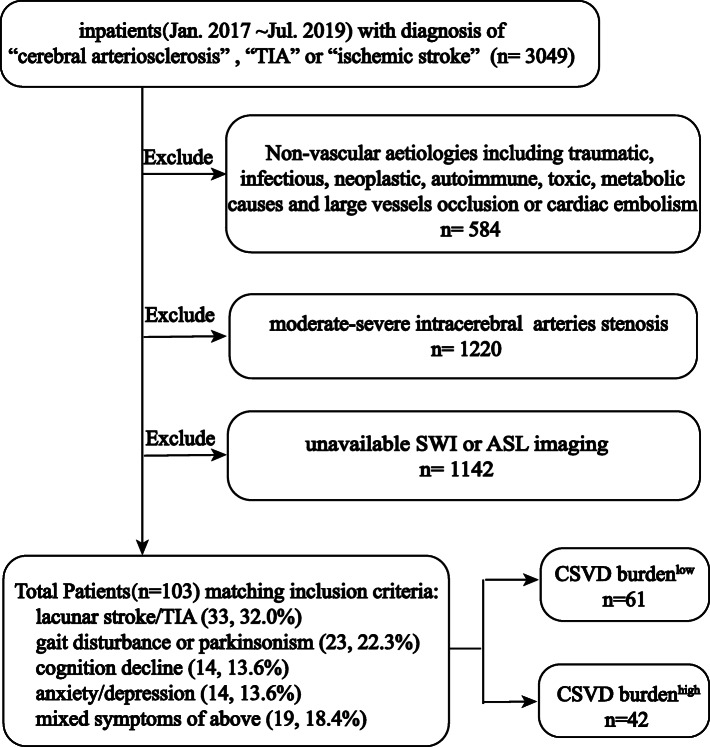
Patients inclusion flow chart

One hundred and three patients (inclusion workflow shown in Fig. 1) admitted to neurology department in Third Affiliated Hospital of Sun Yat-sen University from Sep. 2017 to Dec. 2019 matched the inclusion criteria below: 1) age ≥ 40y; 2) at least one of the following atherosclerotic risk factors: smoking (≥10 cigarettes/day for at least 10y), excessive alcohol consumption (≥15 drinks per week for men and ≥ 8 drinks per week for women), body mass index (BMI)>25, hypertension, diabetes mellitus, impaired glucose tolerance (IGT) or impaired fasting glucose (IFG), coronary heart disease, hyperlipemia, hyperhomocysteinemia, symptomatic stroke history; 3) magnetic resonance imaging (MRI) confirmed recent subcortical small infarct (RSSI) or complaining of CSVD common symptoms including cognitive decline, gait disturbance or bradykinesia, emotional stress (anxiety or depression) and mixture of the above symptoms. Mini-mental state examination (MMSE) and Montreal cognitive assessment (MoCA) were conducted to evaluate the global cognitive function. The optimal demographic-stratified cutoff point for determining cognitive impairment referred to a previous study in Chinese population [15]. If MMSE result was 24 or above, MoCA was administrated to check for mild cognitive impairment [16]. The symptoms of gait disturbance and bradykinesia were assessed by experienced neurologists according to neurological physical examination. The emotional stress was evaluated by Zung’s self-rating depression scale (SDS) [17] and Zung’s self-rating anxiety scale (SAS) [18]. 4) MR neuroimaging met the STandards for ReportIng Vascular changes on nEuroimaging recommendation [19]. 5) No visible moderate-severe intracranial atherosclerotic stenosis in MR angiography. Non-vascular aetiologies including traumatic, infectious, neoplastic, autoimmune, toxic, metabolic causes and large vessels occlusion or cardiac embolism were all excluded. All included patients completed standard brain MRI including arterial spin labelling (ASL) and 81 patients undergone comprehensive transthoracic echocardiography (TTE). The demographic information, medical history, laboratory biomedical test and auxiliary examinations were reviewed from the electronic medical record system.

### Acquisition of carotid ultrasonographic and echocardiographic data

Carotid ultrasonography was performed for measuring intima media thickness and excluding individuals with stenotic degree more than 50% according to North American Symptomatic Carotid Endarterectomy Trial (NASCET) standard [20]. NASCET standard evaluates the degree of stenosis based on the formula: % stenosis = [[1-(D_stenosis_/D_normal_)] × 100] in which D_stenosis_ denotes the diameter of residual lumen at maximal luminal narrowing and D_normal_ denotes the diameter of normal segment distal to stenotic lesion. Two-dimensional doppler echocardiography was conducted mainly to screen potential cardiac risk factors or cardiogenic emboli. Only 81 echocardiographic records could be retrieved in hospital information system. We collected echocardiographic parameters including aortic root diameter (ARD), left atrial diameter (LAD), right atrial diameter (RAD), left ventricular end-diastolic diameter (LVEDD), right ventricular diameter (RVD), interventricular septal thickness (IVS), left ventricular posterior wall (LVPW), pulmonary artery diameter (PAD), left ventricular fraction shortening (LVFS), left ventricular ejection fraction (LVEF), transmitral early-diastolic peak velocity wave (E wave), transmitral atrial wave velocity (A wave) and E/A ratio.

### MRI protocol and neuroimaging assessment

MRI was performed on a GE 3.0-T scanner (MR750, General Electric, Milwaukee, USA) with a standard 8-channels HRBRAIN coil. The MRI protocol included: 1) axial T1 weighted: repetition time (TR) = 1750 ms, echo time (TE) = 24 ms, echo train length (ETL) = 10, bandwidth (BW) = 41.67KHz, matrix = 320 × 224, filed-of-view (FOV) = 240 mm, slice thickness = 5 mm, spacing = 1, number of excitations (NEX) = 1; 2) axial T2 periodically rotated overlapping parallel lines with enhanced reconstruction (POPELLER) weighted fast recover fast spin echo (FrFSE): TR = 5727 ms, TE = 93 ms, ETL = 32, BW = 83.3KHz, matrix = 512 × 512, FOV = 240 mm, slice thickness = 5 mm, spacing = 1, NEX = 1.5; 3) T2 fluid-attenuated inversion recovery (FLAIR) weighted imaging: TR = 8400 ms, TE = 145 ms, inversion time (TI) = 2100 ms, BW = 83.3KHz, flip angle (FA) = 145°, matrix = 320 × 224, FOV = 240 mm, slice thickness = 5 mm, spacing = 1, NEX = 1; 4) axial 3-diameteral time-of flight MR angiography (3D-TOF MRA): TR = 25 ms, TE = 3.4 ms, FA = 20°, BW = 41.67KHz, matrix size = 384 × 320, FOV = 200 mm, slice thickness = 0.8 mm, NEX = 1; 5) axial T2* weighted angiography (SWAN): TR = 77.3 ms, TE = 45 ms, BW = 62.5KHz, FA = 15°, matrix = 384 × 320, slice thickness = 1 mm, NEX = 1; 6) axial 3D ASL: TR 4802 ms, TE 10.5 ms, TI = 2025 ms, BW = 62.5KHz, NEX = 3, FOV = 240 mm, matrix = 512 × 8.

MRI data was analysed by experienced neuroradiologists (Guo N, Zhang K) blinded to clinical information in Functool software on GE AW workstation. We focused on deep regional CBF given that penetrating small vessels lack of collateral circulation were more vulnerable to cerebral hypoperfusion. For CBF analysis, a rectangular region of interest (ROI) with 1 × 4 cm area was delineated symmetrically in the deep regions of both hemispheres on five consecutive planes including centrum semiovale, roof of lateral ventricles, body of third ventricle, upper level of third ventricle and lower level of third ventricle. Average CBF of both hemispheric deep regions on the same plane was recorded as the deep regional CBF of each plane. Finally, the average CBF of five planes was defined as the mean deep regional CBF. Based on the definitions and imaging principal summarized in STandards for ReportIng Vascular changes on nEuroimaging [19], total CSVD neuroimaging burden was assessed according to an ordinal CSVD score (0–4) [21]. One point was scored if neuroimaging matched each of the following four categories: one or more lacunae; one or more CMBs; moderate to severe basal ganglia perivascular space (BG-PVS) [22]; periventricular WMH Fazekas scale 3(extending into the deep white matter) or deep WMH Fazekas scale 2 ~ 3(early confluent or confluent) [5]. Additionally, cerebral atrophy was scored according to global cortex atrophy (GCA) rating scale from 0 to 3 (0 = absent, 1 = mild, 2 = moderate, 3 = severe) [23]. Finally, we dichotomized total 103 patients as CSVD burden^low^ (CSVD burden score 0 ~ 1) and CSVD burden^high^ (CSVD burden score 2 ~ 4). MRI scanning and the aforementioned ultrasonographic examinations were conducted during the same hospitalization.

### Statistical analysis

Data were reported as mean ± standard deviation (SD) for normally distributed variables, median (interquartile range, IQR) for skewedly distributed quantitative variables respectively and numbers (percentages) for qualitative variables. Univariate analysis of clinical factors between patients with CSVD burden^low^ and CSVD burden^high^ was assessed by Student’s t tests or Mann-Whitney U tests depending on variables distribution. Pearson χ^2^ test was used for categorical variables comparison. Multivariate linear regression analysis was performed to determine the contribution of clinical and echocardiographic parameters to mean deep CBF. Backward stepwise (likelihood ratio) binary logistic regression model was constructed to determine factors associated with higher CSVD neuroimaging burden. Statistical analysis was performed in SPSS 25.0 (IBM, Armonk, New York) and *P*<0.05 was considered of statistical significance.

## Results

### Clinical characteristics

In total, 103 patients including 64 males (62.1%, mean age 63.00 ± 11.57 year) and 39 females (37.9%, mean age 63.56 ± 12.27 year) were included into the present study. The primary causes for admission were briefly listed as follow: “lacunar stroke” (33, 32.0%), “gait disturbance or bradykinesia” (23, 22.3%), “cognition decline” (14, 13.6%), “anxiety or depression” (14, 13.6%), mixed symptoms of above (19, 18.4%). The clinical characteristics including demographic information and laboratory biomedical indexes between CSVD burden^low^ and CSVD burden^high^ groups were summarized in Table 1. Patients with CSVD burden^high^ had higher prevalence of hypertension, diabetes mellitus and symptomatic stroke accompanied with higher fasting plasma glucose but lower eGFR compared to patients with CSVD burden^low^.

**Table 1:** Univariate analysis for clinical profiles of CSVD burden^low^ and CSVD burden^high^ groups

	CSVD burden^low^ n = 61	CSVD burden^high^ n = 42	*P*
Demographics			
Age, y	61.53 ± 10.71	65.97 ± 13.23	0.063
Male, n (%)	35 (57.4%)	29 (69.0%)	0.230
Smoking, n (%)	19 (31.1%)	10 (23.8%)	0.416
Alcohol Drinking, n (%)	5 (8.2%)	0 (0.0%)	0.151
BMI	23.04 ± 3.40	24.46 ± 3.23	0.054
Medical History, n (%)			
Hypertension	33 (54.1%)	38 (90.5%)	**< 0 .001**
Impaired Fasting Glucose	1 (1.6%)	1 (2.4%)	1.000
Impaired Glucose Tolerance	4 (6.6%)	3 (7.1%)	1.000
Diabetes Mellitus	10 (16.4%)	16 (38.1%)	**0.013**
Hyperlipidemia	37 (60.7%)	25 (59.5%)	0.908
Atrial Fibrillation	1 (1.6%)	1 (2.4%)	1.000
Previous myocardial infarction	2 (3.3%)	4 (9.5%)	0.184
Symptomatic Stroke	27 (44.3%)	28 (66.7%)	**0.025**
Medication History, n (%)			
Antiplatelet therapy	7 (11.5%)	5 (11.9%)	1.000
Statins	5 (8.2%)	6 (14.3%)	0.510
Antihypertensive Drugs	18 (29.5%)	25 (59.5%)	0.002
Antidiabetic Drugs	8 (13.1%)	11 (26.2%)	0.930
Metabolic Variables^a^			
Fasting Glucose (mmol/L)	5.08 (1.16)	5.41 (1.03)	**0.024**
HbA1c (%)	5.60 (0.67)	5.85 (0.80)	0.493
Total Cholesterol (mmol/L)	4.82 ± 1.17	4.48 ± 1.37	0.434
Triglyceride (mmol/L)	1.32 (0.67)	1.28 (0.93)	0.511
HDL-C (mmol/L)	1.20 ± 0.32	1.13 ± 0.33	0.365
LDL-C (mmol/L)	3.03 ± 0.92	2.71 ± 1.04	0.400
ApoA1 (g/L)	1.32 ± 0.26	1.30 ± 0.26	0.887
ApoB 100 (g/L)	0.98 (0.42)	0.89 (0.53)	0.430
Lp(a) (mg/L)	146.50 (273.00)	137.50 (181.75)	0.957
Homocysteine (μmol/L)	13.21 (6.63)	14.44 (8.40)	0.653
Vitamin B1 (nmol/L)	67.28 (38.04)	68.69 (27.82)	0.407
Vitamin B6(μmol/L)	19.45 (8.02)	19.26 (7.57)	0.957
Vitamin B12(pg/mL)	248.27 (70.93)	239.08 (77.11)	0.330
25-(OH) Vitamin D (nmol/L)	66.90 ± 19.42	62.11 ± 18.15	0.154
eGFR (ml/min/1.73m^2^)	90.45 (19.18)	77.79 (34.07)	**0.039**
Intima Media Thickness (mm)	0.870 ± 0.166	0.875 ± 0.170	0.896
Echocardiographic Parameters			
LAD (mm)	30.76 ± 3.45	32.40 ± 3.38	**0.036**
LVEDV (ml)	90.58 ± 19.24	96.16 ± 17.03	0.179
RVD (mm)	20.07 ± 3.49	21.06 ± 2.81	0.172
RAD (mm)	41.26 ± 4.94	41.86 ± 4.31	0.571
LVFS (%)	37.89 ± 3.47	37.46 ± 4.53	0.626
E/A Ratio	0.86 ± 0.30	0.76 ± 0.22	0.080
LVEF (%)	67.91 ± 4.69	67.26 ± 5.49	0.654

^a^Results were presented as mean ± SD for normally distributed continuous variables or median (interquartile range, IQR) for skewedly distributed continuous variables

*Abbreviations*: *BMI* body mass index, *HbA1c* glycated haemoglobin A1c, *LDL-C* low density lipoprotein-cholesterol, *HDL-C* high density lipoprotein-cholesterol, *ApoA1* apolipoprotein A1, *ApoB100* apolipoprotein B100, *Lp(a)* lipoprotein (a), *eGFR* estimated glomerular filtration rate, *LAD* left atrial diameter, *LVEDV* left ventricular end-diastolic volume, *RVD* right ventricular diameter, *RAD* right atrial diameter, *LVFS* left ventricular fraction shortening, *E/A ratio* [transmitral early-diastolic peak velocity wave (E wave)]/ [transmitral atrial wave velocity (A wave)] ratio, *LVEF* left ventricular ejection fraction

### Univariate analysis for deep regional CBF associated with CSVD burden

In univariate analysis as shown in Table 2, decreased CBF in centrum semiovale (*OR* 0.923; *95% CI* 0.853–0.998; *P* = 0.044) and roof of lateral ventricle (*OR* 0.910; *95% CI* 0.835–0.991; *P* = 0.031) were significant risk factors of CSVD burden^high^.

**Table 2:** Univariate analysis for deep regional CBF associated with CSVD burden

Deep regional CBF (ml/100 g/min)	CSVD burden^low^	CSVD burden^high^	OR (95% CI)	*P*
	(*n* = 46)	(*n* = 35)		
Centrum semiovale	29.45 ± 6.77	26.93 ± 4.42	0.923 (0.853–0.998)	**0.044**
Roof of lateral ventricles	29.86 ± 5.92	27.45 ± 4.25	0.910 (0.835–0.991)	**0.031**
Body of lateral ventricles	32.10 ± 6.67	29.76 ± 4.74	0.930 (0.863–1.002)	0.058
Upper of third ventricle	33.77 ± 7.23	32.28 ± 4.96	0.962 (0.900–1.028)	0.250
Lower of third ventricle	36.98 ± 7.67	35.85 ± 4.91	0.974 (0.915–1.037)	0.402

### Univariate linear regression analysis and correlation matrix of deep regional CBF

**Fig. 2:**
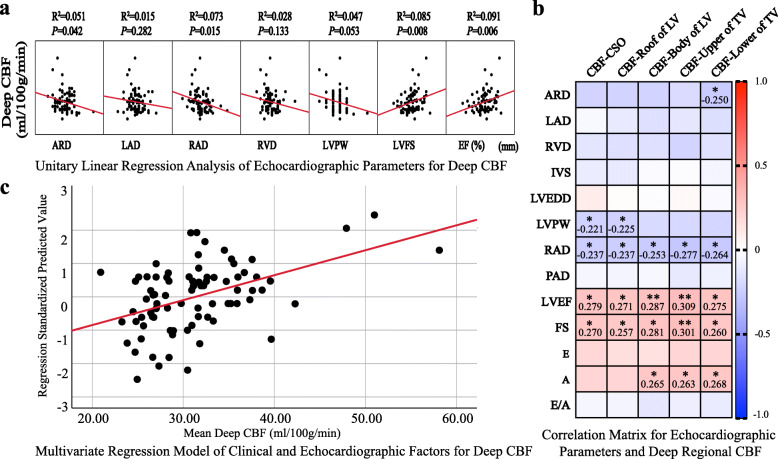
Linear regression analysis and Pearson correlation matrix analysis of deep regional CBF **a**: Unitary linear regression analysis of echocardiographic parameters and mean deep regional CBF **b**: Pearson correlation matrix of echocardiographic parameters and five deep regional CBF (number shown in the matrix is *r* coefficient with statistical significance, * for *P* < 0.05 and ** for *P* < 0.01). **c**: Multilinear regression model of clinical and echocardiographic factors for deep regional CBF (*R*^*2*^ = 0.328, *P* = 0.002 for multiple linear regression equation)

The average of five planes of deep regional CBF was defined as the “mean deep regional CBF”. Unitary linear regression analysis of echocardiographic parameters and mean deep CBF as shown in Fig. 2a demonstrated that aortic root diameter (*B coefficient*, − 0.463; *95% CI*, − 0.908 to − 0.018; *P* = 0.042), RAD (*B coefficient*, − 0.356; *95% CI*, − 0.640 to − 0.072; *P* = 0.015), LVEF (*B coefficient* 36.846; *95% CI*, 10.796 to 62.895; *P* = 0.006) and LVFS (*B coefficient*, 45.266; *95% CI*, 11.912 to 78.619; *P* = 0.008) were linearly associated with mean deep CBF. Pearson correlation matrix as shown in Fig. 2b revealed LVEF (r = 0.279, 0.271, 0.287, 0.309, 0.275 and *P* = 0.012, 0.014, 0.009, 0.005, 0.013 respectively) and LVFS (*r =* 0.270, 0.257, 0.281, 0.301, 0.260 and *P* = 0.015, 0.020, 0.011, 0.006, 0.019 respectively) were positively correlated with five deep regional CBF while RAD (*r* = − 0.237, − 0.237, − 0.253, − 0.277, − 0.264 and *P* = 0.033, 0.033, 0.023, 0.012, 0.017 respectively) was negatively correlated with each plane of deep regional CBF.

### Multivariate linear regression analysis for factors associated with deep regional CBF

In multivariate linear regression analysis as shown in Table 3, in addition to hypertension (*B coefficient*, 3.141; *95% CI*, 0.201 to 6.080; *P* = 0.037), RAD (*B coefficient*, − 0.289; *95% CI*, − 0.578 to − 0.001; *P* = 0.049) and LVEF (*B coefficient*, 32.555; *95% CI*, 7.399 to 57.711; *P* = 0.012) were structural and functional echocardiographic parameters linked with deep regional CBF in CSVD patients respectively. As shown in Fig. 2c, multivariate linear regression model demonstrated predictive significance of clinical risk factors and echocardiographic parameters for evaluating mean deep CBF (*R*^*2*^ = 0.328, *P* = 0.002). Variance inflation factors of all included variables were less than 1.85, indicating no collinearity among included variables in the regression model.

**Table 3:** Multivariate linear regression analysis for factors associated with mean deep regional CBF

Clinical and Echocardiographic Factors	***B*** (95% CI)	***β***	***P***
Constant variable	26.060 (−3.750 to 55.870)		0.086
Age, y	0.860 (−0.042 to 0.215)	0.162	0.185
Hypertension	3.141 (0.201 to 6.080)	0.235	**0.037**
Symptomatic stroke	−1.520 (− 4.331 to 1.291)	−0.122	0.285
eGFR (ml/min/1.73m^2^)	0.060 (−0.013 to 0.134)	0.200	0.107
Aortic root diameter (mm)	−0.405 (− 0.848 to 0.038)	−0.198	0.072
Left ventricular posterior wall (mm)	−1.207 (−2.706 to 0.292)	−0.207	0.113
Left ventricular end-diastolic diameter (mm)	0.191 (−0.157 to 0.539)	0.119	0.277
Interventricular septal (mm)	0.595 (−0.249 to 1.439)	0.187	0.164
Right atrial diameter (mm)	− 0.289 (− 0.578 to − 0.001)	−0.219	**0.049**
Right ventricular diameter (mm)	−0.410 (− 0.822 to 0.001)	−0.216	0.051
Left ventricular ejection fraction (%)	32.555 (7.399 to 57.711)	0.267	**0.012**

*Abbreviation*: *eGFR* estimated glomerular filtration rate

*R*^*2*^ = 0.328, *P* = 0.002 for multivariate linear regression equation

### Correlation matrix analysis of deep regional CBF, echocardiographic parameters and CSVD neuroimaging markers

**Fig. 3:**
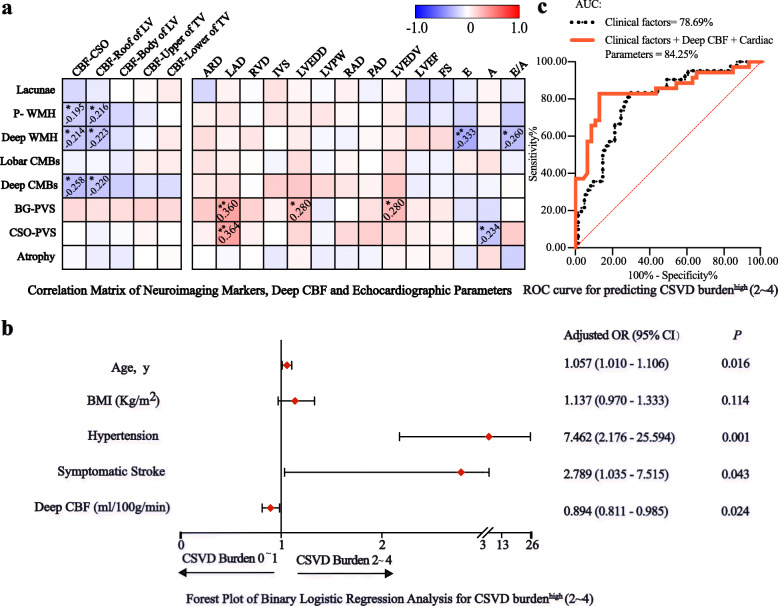
Correlation analysis for deep regional CBF, echocardiographic parameters, neuroimaging markers and multivariate regression analysis for factors associated with CSVD burden^high^ **a** Spearman correlation matrix of CSVD radiological markers, deep regional CBF and echocardiographic parameters (number shown in the matrix is *r* coefficient with statistical significance, * for *P* < 0.05 and ** for *P* < 0.01) **b** Forest plot of binary logistic regression analysis for CSVD burden^high^ (covariables including age, body mass index, hypertension, symptomatic stroke, diabetes, fasting plasma glucose and eGFR) **c** ROC curve for predicting CSVD burden^high^ Black dashed line denotes ROC curve analysed with clinical risk factors including age, hypertension, symptomatic stroke and eGFR; red solid line denotes ROC curve analysed with clinical risk, mean deep regional CBF and echocardiographic parameters including ARD, RVD, IVS, LVEDD, LVPW, RAD, PAD, LVEF, FS and E/A ratio

Spearman correlation matrix as shown in Fig. 3a demonstrated that both CBF in centrum semiovale and roof of lateral ventricle were negatively correlated with periventricular WMH (*r*_*s*_ = − 0.195, *P* = 0.048; *r*_*s*_ = − 0.216, *P* = 0.028 respectively), deep WMH (*r*_*s*_ = − 0.214, *P* = 0.030; *r*_*s*_ = − 0.223, *P* = 0.023 respectively) and deep CMBs (*r*_*s*_ = − 0.258, *P* = 0.009; *r*_*s*_ = − 0.220, *P* = 0.025 respectively). Besides, left atrial diameter positively correlated with BG-PVS (*r*_*s*_ = 0.360, *P* = 0.001) and centrum semiovale-PVS (*r*_*s*_ = 0.364, *P* = 0.001. Left ventricular end-diastolic volume/diameter were positively correlated with numbers of BG-PVS (*r*_*s*_ = 0.280, *P* = 0.011). Transmitral early-diastolic wave (E wave) velocity (*r*_*s*_ = − 0.333, *P* = 0.002) and E/A ratio (*r*_*s*_ = − 0.260, *P* = 0.019) were negatively correlated with deep WMH.

### Multivariate analysis of attributable factors for higher CSVD burden

In binary logistic regression analysis as shown in Fig. 3b, mean deep CBF (*OR* 0.894; *95% CI* 0.811–0.985; *P* = 0.024) was independently associated with higher CSVD burden after adjusted for clinical risk factors including hypertension (*OR* 7.426; *95% CI* 2.176–25.594; *P* = 0.001), symptomatic stroke (*OR* 2.789; *95% CI* 1.035–7.515; *P* = 0.043) and age (*OR* 1.057; *95% CI* 1.010 to 1.106, *P* = 0.016). Furthermore, receiver operating characteristics (ROC) curve integrating clinical profiles (hypertension, age, symptomatic stroke and elevated eGFR), mean deep CBF and echocardiographic parameters (ARD, RVD, IVS, LVEDD, LVPW, RAD, PAD, LVEF, FS and E/A) showed higher predictive significance for CSVD burden^high^ (*AUC* = 84.25, *95% CI* 74.86–93.65%, *P* < 0.0001) compared to ROC curve introduced with clinical risk factors (*AUC* = 78.69, *95% CI 69.75–87.63%, P* < 0.0001) (Fig. 3c).

## Discussion

In the present study, we observe a linear relationship between RAD, LVEF and mean deep regional CBF whose decline contributes to higher CSVD neuroimaging burden. We propose that echocardiographic and deep regional CBF assessment should be attached importance in the prognostic evaluation of aCSVD patients absent from heart failure.

Previous study indicates that lower LVEF prolongs arterial input function and contributes to larger hypoperfusion volumes as well as poorer poststroke outcome [24]. Though the present study enrolled an aCSVD population with normal LVEF, we have revealed an independent positive linear correlation between deep regional CBF and LVEF after adjustment of conventional cerebrovascular risk factors including age, hypertension, symptomatic stroke and eGFR. Besides, deep parenchymal territorial is believed to be more susceptible to microcirculation hypoperfusion due to lack of sufficient collateral circulation. Hence, we supposed that deep regional CBF was particularly vulnerable to normal but lower LVEF. In addition, LVFS another index of left ventricular systolic function was positively associated with deep regional CBF. Contrarily, no significant correlation was found between E/A ratio and deep regional CBF, indicating left ventricular systolic function but not diastolic function tipped the balance in deep regional perfusion.

Recent systemic review and meta-analysis confirms global CBF reduction in CSVD patients especially for those with WMH [25]. Furthermore, global hypoperfusion is linked with BBB leakage in CSVD patients with WMH or normal-appearing white matter [6]. BBB leakage followed by neurovascular unit (NVU) inflammation and energy metabolic disturbance cascade is thereby considered as a predominant pathogenesis of aCSVD [1, 26]. It is worth noting that the present study focused on deep medullary territories CBF supply instead of global CBF in aCSVD population absent from chronic heart failure. The small penetrating vessels are the main CBF supply of deep parenchyma whose ischemia are predisposed to the development of WMH, CMBs and PVS. In accordance with the correlation between deep CBF in broader-zone regions (centrum semiovale level and roof of lateral ventricles level) and the burden of WMH, deep CMBs, mean deep regional CBF was independently linked with higher CSVD burden.

Notably, RAD was negatively correlated with mean deep regional CBF. The association between right atrium enlargement and CBF perfusion has seldom been elucidated previously. It has been confirmed that right atrium enlargement is usually in parallel with elevated right atrial pressure [27]. Moreover, elevated right atrial pressure indicates increased cerebrovascular resistance and cerebrovenous congestion [28]. It is supposed that dilated right atrium may indirectly reflect elevated right atrial pressure which impedes efflux of glymphatic drainage from PVS. Inadequate glymphatic clearance may ultimately contribute to accumulation of toxic metabolic by-products and subsequent NVU inflammation [29]. Right atrial hemodynamics involved aCSVD pathogenesis is supported by the evidence that mean right atrial pressure is independently associated with higher WMH volume in chronic valvular heart disease [30]. Collectively, cardiac structural and functional remodelling participates in aCSVD development via complicated “heart-brain axis” as shown in Supplementary Fig. 1.

It seems no direct correlation between LVEF with CSVD neuroimaging markers and the relevant reasons are summarized below. Firstly, patients with cardiac embolism and moderate-severe intracranial atherosclerotic stenosis were excluded and all participants maintained normal LVEF in the present study. It is supposed that the cerebral perfusion and CSVD neuroimaging markers are more vulnerable to cardiac hemodynamics alteration in chronic heart failure but not in normal heart function state. Actually, the patients with chronic heart failure would more remarkably benefit from increased cardiac perfusion than those with normal cardiac function [31]. Secondarily, though increased LVEF may increase the CBF supply in aCSVD patients, persistently enhanced cardiac contraction and arterial pulsatility may concomitantly contribute to cerebrovascular remodelling and elevation of cerebrovascular resistance [32]. Subsequently, long-term effect of cerebral hemodynamic stress may counteract the modest protective effect from subtle elevated LVEF in aCSVD patients absent from heart failure. Collectively, the cardiac regulation of cerebral hemodynamics is a complicated integrated effect [33] and further studies stratified heart function grading are warranted to explore the cardiac hemodynamic effects on CSVD burden.

There are some limitations in our study. Firstly, the hemodynamic data from cardiac catheterization was not available. Compared to transthoracic echocardiography, cardiac catheterization data is more convincing to elucidate the link between cardiac and cerebral hemodynamics. However, it is reasonable and reliable for aCSVD patients to assess cardiac structure and function via echocardiography instead of cardiac catheterization given the invasive operational risk and medical cost. Secondarily, it is a cross-sectional study lack of follow-up data to validate the causality relation between cardiac remodelling and deep regional CBF.

## Conclusion

In conclusion, LVEF and RAD are functional and structural echocardiographic parameters modifying deep regional CBF whose decline indicates poor microcirculation perfusion and higher CSVD burden. Feasible echocardiographic and deep regional CBF assessment will provide prognostic significance for the early-warning of high CSVD burden.

## Supplementary Information

**Additional file 1: Supplementary Fig. 1.** Schematic of “heart-brain axis” hypothesis Firstly, chronic arteriolosclerosis contributes to long-term microcirculation ischemia. Furthermore, lower LVEF results in declined small vessel CBF supply. Secondarily, enlargement of right atrium suggestive of increased cerebral venous return resistance decreases cerebral interstitial fluid return. Finally, the insufficient para-arterial influx and para-venous efflux contributes to inadequate glymphatic clearance and PVS inflammation. The subsequent blood-brain barrier disfunction exacerbates CSVD neuroimaging burden. Abbreviations: CBF, cerebral blood flow; LVEF, left ventricular ejection fraction; RAD, right atrial diameter; PA pulmonary artery; SCV, subclavian vein; SCA, subclavian artery; CCA, common carotid artery; SVC, superior vena cava; CJV, cervical jugular vein; PVS, perivascular space; BBB, blood brain barrier.

## Data Availability

The datasets used and analysed during the current study are available from the corresponding author on reasonable request.
