# Supplementary material for: Left ventricular ejection fraction and right atrial diameter are associated with deep regional CBF in arteriosclerotic cerebral small vessel disease
Source: BMC Neurol. 2021 Feb 11;21:67. doi: 10.1186/s12883-021-02096-w (PMC7877009; doi:10.1186/s12883-021-02096-w)
Supplement: Supplementary file 1 — Additional file 1: Supplementary Fig. 1. Schematic of “heart-brain axis” hypothesis Firstly, chronic arteriolosclerosis contributes to long-term microcirculation ischemia. Furthermore, lower LVEF results in declined small vessel CBF supply. Secondarily, enlargement of right atrium suggestive of increased cerebral venous return resistance decreases cerebral interstitial fluid return. Finally, the insufficient para-arterial influx and para-venous efflux contributes to inadequate glymphatic clearance and PVS inflammation. The subsequent blood-brain barrier disfunction exacerbates CSVD neuroimaging burden. Abbreviations: CBF, cerebral blood flow; LVEF, left ventricular ejection fraction; RAD, right atrial diameter; PA pulmonary artery; SCV, subclavian vein; SCA, subclavian artery; CCA, common carotid artery; SVC, superior vena cava; CJV, cervical jugular vein; PVS, perivascular space; BBB, blood brain barrier. [file 12883_2021_2096_MOESM1_ESM.pdf]

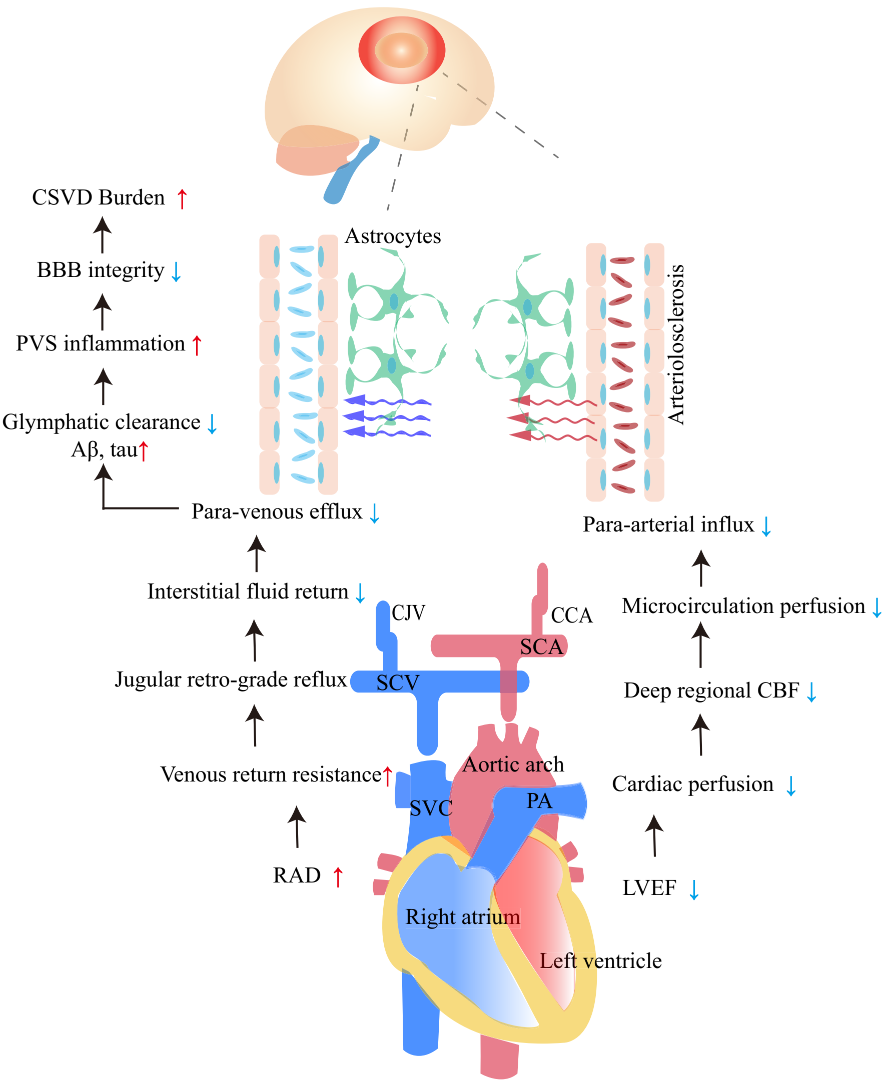

Schematic of “heart-brain axis” hypothesis

Supplementary Fig. 1 Schematic of “heart-brain axis” hypothesis Firstly, chronic arteriolosclerosis contributes to long-term microcirculation ischemia. Furthermore, lower LVEF results in declined small vessel CBF supply. Secondarily, enlargement of right atrium suggestive of increased cerebral venous return resistance decreases cerebral interstitial fluid return. Finally, the insufficient para-arterial influx and para-venous efflux contributes to inadequate glymphatic clearance and PVS inflammation. The subsequent blood-brain barrier disfunction exacerbates CSVD neuroimaging burden. Abbreviations: CBF, cerebral blood flow; LVEF, left ventricular ejection fraction; RAD, right atrial diameter; PA pulmonary artery; SCV, subclavian vein; SCA, subclavian artery; CCA, common carotid artery; SCV, superior vena cava; CJV, cervical jugular vein; PVS, perivascular space; BBB, blood brain barrier
